# Unraveling the prognostic significance and molecular characteristics of tumor-infiltrating B lymphocytes in clear cell renal cell carcinoma through a comprehensive bioinformatics analysis

**DOI:** 10.3389/fimmu.2023.1238312

**Published:** 2023-10-16

**Authors:** Youwei Yue, Xinyi Cai, Changhao Lu, Leonardo Antonio Sechi, Paolo Solla, Shensuo Li

**Affiliations:** ^1^ Department of Urology, Longgang District Central Hospital of Shenzhen, Shenzhen, China; ^2^ Department of Pathology, Provincial Key Laboratory of Infectious Diseases and Molecular Immunopathology, Shantou University Medical College, Shantou, China; ^3^ Department of Biomedical Sciences, University of Sassari, Sassari, Italy; ^4^ Department of Medical, Surgical and Experimental Sciences, University of Sassarie, Sassari, Italy; ^5^ Shanghai Frontiers Science Center for Chinese Medicine Chemical Biology, Institute of Interdisciplinary Integrative Medicine Research, Shanghai University of Traditional Chinese Medicine, Shanghai, China

**Keywords:** clear cell renal cell carcinoma (ccRCC), tumor microenvironment, tumor-infiltrating B lymphocytes (TIL-Bs), prognosis, biomarker, gene signature

## Abstract

**Introduction:**

Clear cell renal cell carcinoma (ccRCC) is a prevalent subtype of kidney cancer that exhibits a complex tumor microenvironment, which significantly influences tumor progression and immunotherapy response. In recent years, emerging evidence has underscored the involvement of tumor-infiltrating B lymphocytes (TIL-Bs), a crucial component of adaptive immunity, and their roles in ccRCC as compared to other tumors. Therefore, the present study endeavors to systematically explore the prognostic and molecular features of TIL-Bs in ccRCC.

**Methods:**

Initially, xCell algorithm was used to predict TIL-Bs in TCGA-KIRC and other ccRCC transcriptomic datasets. The Log-Rank test and Cox regression were applied to explore the relationship of B-cells with ccRCC survival. Then, we used WGCNA method to identify important modules related to TIL-Bs combining Consensus subcluster and scRNA-seq data analysis. To narrow down the prospective biomarkers, a prognostic signature was proposed. Next, we explored the feature of the signature individual genes and the risk-score. Finally, the potential associations of signature with clinical phenotypes and drugs were investigated.

**Results:**

Preliminary, we found ccRCC survival was negatively associated with TIL-Bs, which was confirmed by other datasets. Afterwards, ten co-expression modules were identified and a distinct ccRCC cluster was subsequently detected. Moreover, we assessed the transcriptomic alteration of B-cell in ccRCC and a relevant B-cell subtype was also pinpointed. Based on two core modules (brown, red), a 10-gene signature (TNFSF13B, SHARPIN, B3GAT3, IL2RG, TBC1D10C, STAC3, MICB, LAG3, SMIM29, CTLA4) was developed in train set and validated in test sets. These biomarkers were further investigated with regards to their differential expression and correlation with immune characteristics, along with risk-score related mutations and pathways. Lastly, we established a nomogram combined tumor grade and discovered underlying drugs according to their sensitivity response.

**Discussion:**

In our research, we elucidated the remarkable association between ccRCC and B-cells. Then, we detected several key gene modules, together with close patient subcluster and B-cell subtype,which could be responsible for the TIL-Bs in ccRCC. Moreover, we proposed a 10-gene signature and investigated its molecular features from multiple perspectives. Overall, understanding the roles of TIL-Bs could aid in the immunotherapeutic approaches for ccRCC, which deserve further research to clarify the implications for patient prognosis and treatment.

## 1 Introduction

Kidney cancer is a prevalent malignant neoplasm globally, particularly in Europe and North America, with an estimated over 400,000 new cases diagnosed in 2020 (1, 2). Renal cell carcinoma (RCC), which arises in the renal cortex, accounts for approximately 90% of all primary kidney neoplasms and encompasses histological subtypes included clear cell RCC (ccRCC), papillary RCC (pRCC), and chromophobe RCC (chRCC) (3). Of these subtypes, ccRCC is the predominant form (~80%) and exhibits a higher propensity for metastasis and worse prognostic outcomes (4, 5). Given the high frequency of recurrence and adverse reactions, clinical research and drug discovery have shifted their focus towards therapies that target key oncogenic genes and pathways, or activate the anti-tumor response of the immune system (6, 7). For example, VHL is a well-known tumor suppressor gene that is frequently mutated (~70%) in ccRCC (8). It has been demonstrated to regulate the expression of the HIF/VEGF axis, which contributes to angiogenesis and neovascularization. Consequently, target agents such as tyrosine kinase inhibitors (TKIs) have been developed to inhibit VEGFR activity and are widely employed in the treatment of ccRCC (9).

ccRCC is commonly recognized as a hot tumor with a mixed immune microenvironment, leading to the emergence of immunotherapy as a promising therapeutic approach in clinical settings (10). Initially, cytokines, such as interleukin-2 (IL-2), were utilized to directly stimulate the immune system, but high doses could also result in severe side effects (11). In recent years, immunotherapy has shifted towards immune checkpoint inhibitors (ICIs) that target checkpoints (e.g., PD-1, PD-L1, CTLA-4) and aid in restoring the immune response against kidney cancer cells (12). They are frequently employed in conjunction with other targeted therapies, such as TKIs for better clinical outcomes. For example, Pembrolizumab, which targets PD-1, may be administered with Axitinib as an initial treatment for individuals with advanced ccRCC (13). Nevertheless, the efficacy of immunotherapy may vary among patients due to numerous potential factors, including the considerable heterogeneity of the tumor microenvironment (TME) in ccRCC (10).

Recently, significant advancements have been achieved in comprehending the intricate condition of immune cell infiltration in ccRCC, owing to the swift progress of single-cell RNA sequencing (scRNA-seq) technology and cutting-edge bioinformatics algorithms (14–16). For instance, Aleksandar Obradovic and his colleagues acquired expression data from over 100,000 cells derived from 11 treatment-naïve ccRCC samples and identified a macrophage subtype (TREM2+/APOE+/C1Q+) that was linked to tumor recurrence through the VIPER algorithm (14). In another scRNA-seq investigation of ccRCC, it was discovered that tumor epithelia could react to immune cell infiltration and potential connections between endothelial cells and immunotherapy were also revealed (17). Alternatively, immune infiltration algorithms, such as matrix deconvolution and single-sample gene set enrichment analysis (ssGSEA), offer alternative approach to exploring the TME within tumors, utilizing numerous bulk RNA-seq cohorts. Bai et al. utilized the TCGA-KIRC dataset to predict the level of 29 immune-related signatures and observed subtypes with high immune scores were associated with favorable prognoses (18). Additionally, a protective signature regarding ccRCC CD8+ T cell infiltration was proposed as a means of evaluating survival conditions and identifying novel therapy targets (19).

**Figure 1:**
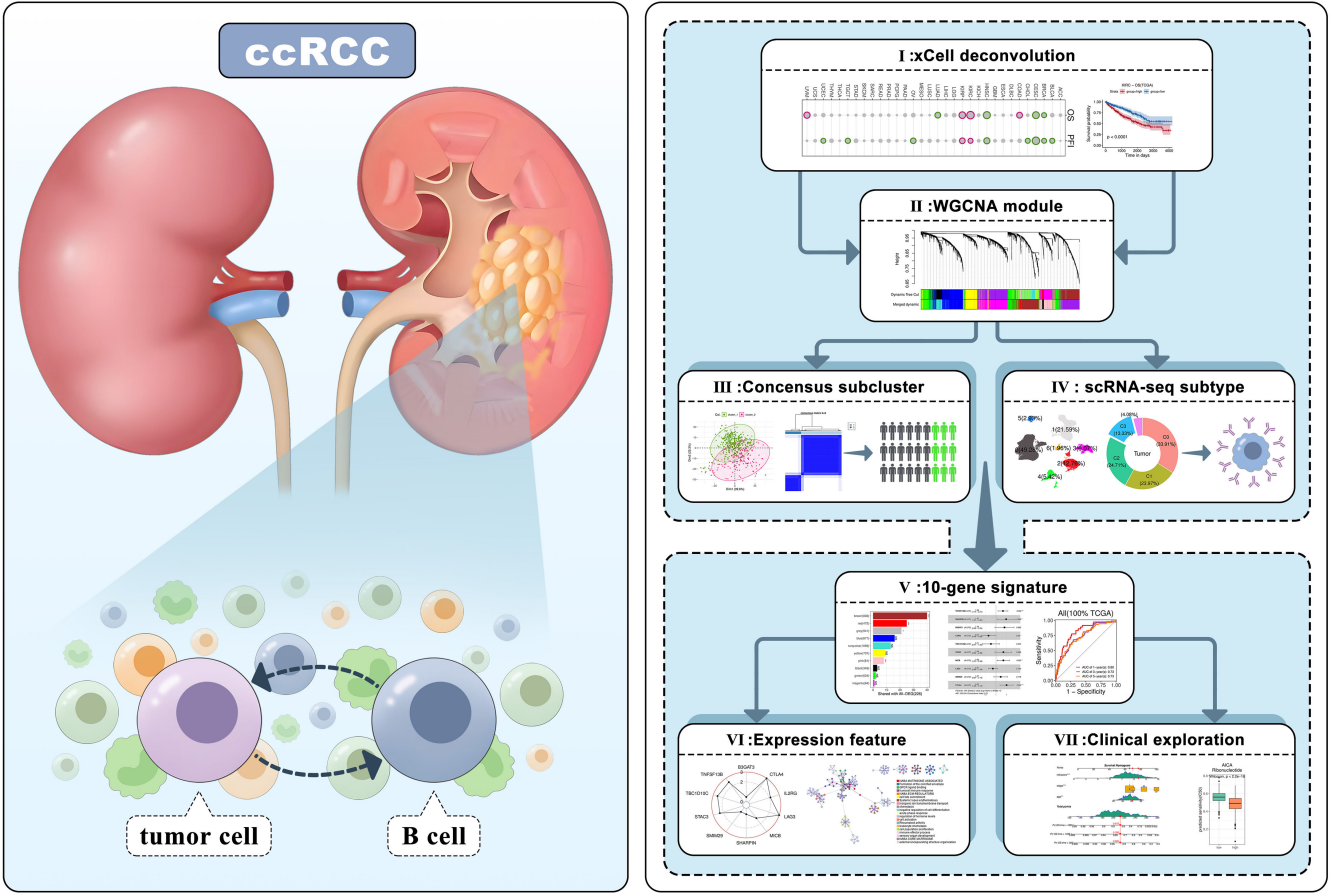
Workflow of the research analytical pipeline.

Nonetheless, research on tumor-infiltrating B lymphocytes (TIL-Bs), encompassing both B cells and plasma cells here, is limited in comparison to other immune cell types. However, their potential roles in the TME cannot be understated, as they may serve as crucial prognostic indicators, biomarkers, and targets for immunotherapy (20, 21). TIL-Bs are predominantly located in tertiary lymphoid structures, alongside T cells, macrophages, etc. Prior investigations have demonstrated that B cells are associated with favorable anti-tumor responses and improved prognoses in various malignancies, including breast (22), colon (23), lung (24), and ovarian cancers (25). Noteworthily, a few studies have suggested that TIL-Bs may have a pro-tumorigenic effect in ccRCC, which differs from their impact on other tumor types (26–28). Therefore, it is imperative to investigate their intricate functions, as this could provide novel insights into the TME of ccRCC and facilitate the development of targeted immune therapies. In this study, we conducted a systematic analysis using multiple datasets and bioinformatics tools to elucidate the underlying characteristics of TIL-Bs in ccRCC (**Figure 1**). Preliminarily, high level of infiltrating B cells was confirmed to be correlated with worse survival in ccRCC. Subsequently, we employed WGCNA to identify important regulatory networks related to TIL-Bs. Based on which, we also identified a ccRCC subtype through Consensus analysis and focused a B-cell subcluster in an scRNA-seq dataset, respectively. According to the hub genes of two core modules, we furtherly screened candidate molecules to construct a 10-gene signature that exhibited satisfactory prognostic efficacy in TCGA train set, as validated by both TCGA test set and ICGC test set. The multifaceted features of the TIL-Bs related signature, such as abnormal expression and immune correlation of model biomarkers, together with close single nucleotide variants (SNVs) and functions of signature based on the risk-score. Finally, a nomogram was developed for more precise prognosis, mainly incorporating tumor stage in clinic. Several prospective drugs against ccRCC patients in high-risk group were also identified.

## 2 Materials and methods

### 2.1 KIRC transcriptomic data collection

The transcriptome expression (529 primary tumor and 72 normal samples) and mutation information (402 samples) of TCGA-KIRC cohort was downloaded through TCGAbiolinks R package, and the corresponding survival and phenotype data were also collected from previous study (29). The ccRCC cohort (RECA-EU) from ICGC Data Portal, together with survival data, were also downloaded. For TCGA and ICGC datasets based on bulk RNA-seq, the FPKM normalization of raw count expression matrix was adopted. Another cohort comprised of 101 ccRCC patients were also obtained from ArrayExpress database, with the accession number of E-MTAB-1980. Several sequencing datasets were also collected from GEO database. There were totally 60 tumor samples involved 4 stages (I, II, III, IV) in GSE150404 dataset, 15 metastasis and 74 primary tumor samples in GSE19949 dataset. GSE126964 dataset focused on Chinese ccRCC with 55 tumor and 11 normal samples. The ccRCC scRNA-seq data of 16 RCC related tumors and 10 controls were obtained based on GSE178481. In our analysis, 10 primary ccRCC tumors and 9 controls not involving metastasis or treatment were selected.

### 2.2 Cell types infiltrating analysis

The xCell algorithm (15), which could infer infiltration scores of common immune cell types, was adopted to predict tissue cellular heterogeneity of samples with normalized bulk RNA-seq or array sequencing data. For TCGA datasets, the xCell results of 33 cancers were directly downloaded from TIMER2.0 website (http://timer.cistrome.org). For other datasets, xCell R package was applied to calculate the immune infiltration. Additionally, we collected nine general marker (BLK, CD19, FCRL2, MS4A1, KIAA0125, TNFRSF17, TCL1A, SPIB, PNOC) that can reflect B-cell population in tumor environment from previous research (30), and all of them were detectable in TCGA datasets except for KIAA0125. The single-sample Gene Set Enrichment Analysis (ssGSEA) method was employed to evaluate the enrichment score of the eight genes by comparing the ranking of them and all genes at individual sample level.

### 2.3 Survival analysis

The two common survival analysis methods, Log-Rank test and Cox proportional hazards regression, were mainly executed and visualized by survival and survminer R package. The Log-Rank test, a non-parametric statistical test, allowed to compare the survival outcomes between groups and assess the significance. Unless otherwise specified, we categorized continuous variables (such as infiltration score, signature risk score) based on their median values in order to facilitate analysis while using the Log-Rank test. On the other hand, Cox regression is a semi-parametric method to evaluate the impact of single factor and multiple covariates on survival and estimate the hazard ratios (HRs). An HR greater than 1 indicates a higher risk with each unit increase, while an HR less than 1 indicates a lower risk.

### 2.4 WGCNA module identification

Weighted Gene Co-expression Network Analysis (WGCNA) was employed to identify the underlying gene networks in ccRCC. Log2 transformed normalized expression matrix of tumor samples from TCGA-KIRC cohort was used as the input data. Then, the top variable genes with higher median absolute deviation (MAD) were selected. Samples with hierarchical clustering distance above 150 were considered as outliers. The signed adjacency co-expression matrix was constructed by calculating robust gene-pairwise correlation and gene modules were identified by hierarchical clustering algorithm. Importantly, the module eigengene (ME) was also obtained for modules to represent their expression patterns. Subsequently, the module membership (MM) of genes was attained by calculating the correlation between gene expression and ME values. The top 30% genes with the highest MM values of each module are considered as hub genes. It is noteworthy that grey module genes are separate from the other clearly defined modules according to WGCNA method.

### 2.5 Consensus cluster analysis

We applied ConsensusClusterPlus R package, a resampling-based clustering algorithm, to discover potential ccRCC subtypes according to hub genes of TIL-Bs related modules. Specifically, the number of subsamples (reps) and proportion of items to sample (pItem) were set to 1000 and 0.8, respectively. The correlation coefficient-based distance matrix was adopted to perform paritioning around medoids (pam) clustering and identify consensus clusters. Then, principal component analysis (PCA) was used to validate the clustering result based on the same input data.

### 2.6 scRNA-seq data analysis

Seurat R package was mainly used to perform scRNA-seq analysis. Firstly, 22,205 genes and 114,216 cells were remained for later analysis after sample selection and quality control (max detected unique genes for cells < 5000, max mitochondria gene expression percentage for cells < 10%; minimum expressed cells for genes >10). Through batch correction by harmony algorithm and clustering analysis (resolution 0.01), we then annotated 7 main cell types by the expression of maker genes (T cell: CD3D, CD3E, CD3G for cluster 0; Myeloid cell:CD68, LYZ, AIF1 for cluster 1; Fibroblast cell:RGS5, BGN, TAGLN for cluster 2; Epithelial cell: KRT18, EPCAM, PAX8 for cluster 3; Endothelial: VWF, CLDN5, FLT1 for cluster 4; B cell: CD79A, CD79B, MS4A1, MZB1 for cluster 5; Mast cell: CPA3, TPSAB1 for cluster 6). In particular, we also obtained 5 subclusters for the B-cell population (resolution 0.5). Then, we identified differentially expressional genes (DEGs) with absolute average log2(Fold change) > 0.2, p-value < 0.05 and calculated the module score for cells using corresponding functions of Seurat package. Besides, we utilized the CellPhoneDB software (31) to assess the significance and expression strength of ligand-receptor pairs between B-cell and other cell types in tumor or normal samples, respectively. Within each group, up to 3000 cells of each main cell type were randomly selected for the cell communication analysis. The B-cell related ligand-receptor pairs with significant enrichment in tumor or normal group through CellPhoneDB analysis were extracted. Scissor method that recently proposed (32) was used to infer survival associated B cells by integrating TCGA-KIRC dataset. Specifically, cell populations marked with “Sccisor +”, which could be detected at specific level resolutions, were thought to be related with poor prognosis.

### 2.7 Prognostic signature and nomogram establishment

The TCGA-KIRC samples with valid survival data were randomly divided into train set (70%) and test set (30%). The independent ICGC dataset was used as external test set. First of all, the FPKM normalized data was dealt with log2 transformation. In the train set, we calculated the HR significance of module hub genes based on univariate Cox regression. Next, the Lasso model was applied to detect redundant features with different regularization parameter lambda values using glmnet R package and the candidate genes were finally confirmed according to lowest partial likelihood deviance. Among these genes, a superior prognostic signature model was proposed and the corresponding risk scores based on the dot product operation of the expression levels of signature genes and their respective HR coefficients. Then, we evaluated the survival prediction efficacy of the score on train set, test set and external set, separately. In details, we grouped each set based on the median risk score and used log-rank test to compare the survival difference. On the other hand, timeROC R package was adopted to calculate the ROC curve of time-dependent predictive performance at 1-, 3-, 5-years and corresponding AUC values. Additionally, nomogram was applied to present the predictions of Cox regression model based on risk score and other clinical factors by converting coefficients into intuitive linear scales, which can be implemented by the rms and regplot R packages.

### 2.8 Gene expression analysis

For DEGs information of TCGA pan-cancer, we directedly extracted from GSCA website (http://bioinfo.life.hust.edu.cn/GSCA), which has analyzed for 14 types of cancers with more than 10 pairs of tumor-normal samples. Their log2(Fold change) values and Wilcox test p-values were calculated based on RSEM normalized mRNA expression data at the platform. For other datasets, we mainly adopted DESeq2 package to identify DEGs based raw count expression matrix of bulk RNA-seq data and limma package to analyze the microarray data under specified grouping condition. Generally, if a gene with its adjusted p-value below 0.01 and absolute log2(Fold change) over 1, it was considered as a significant DEG. For correlation analysis, we calculated the spearman coefficients between signature genes and multiple immune infiltration scores in tumor or normal condition, respectively. Besides, we also obtained 79 immune checkpoint genes (ICs) from previous research (33) and 67 of them that found in our datasets were used to explore correlation with the signature genes.

### 2.9 Pathway enrichment analysis

For the up-regulated and down-regulated genes between ccRCC consensus clusters, we performed enrichment analysis among Reactome pathway database using ClusterProfiler R package, separately. For the pathway related to signature risk-score, Metascape platform (https://metascape.org) was used to annotate top 20 representative biological functions based on significant DEGs. On the other hand, we also applied GSEA algorithm to detect dysregulated hallmark processes collected by MsigDB database (https://www.gsea-msigdb.org/gsea/msigdb).

### 2.10 Drug prediction strategy

The estimating sensitivity response (IC50) of 274 common drugs on TCGA patients was obtained from previous research (34), which were mainly based on cancer cell line data from Genomics of Drug Sensitivity in Cancer (GDSC) Project and CCLE database. Then, the correlation between risk scores of patients and corresponding IC50 values of a drug was calculated. The candidate drugs with significantly negative coefficients were pinpointed.

## 3 Results

### 3.1 Pan-cancer survival analysis based on B cell infiltrating level

**Figure 2:**
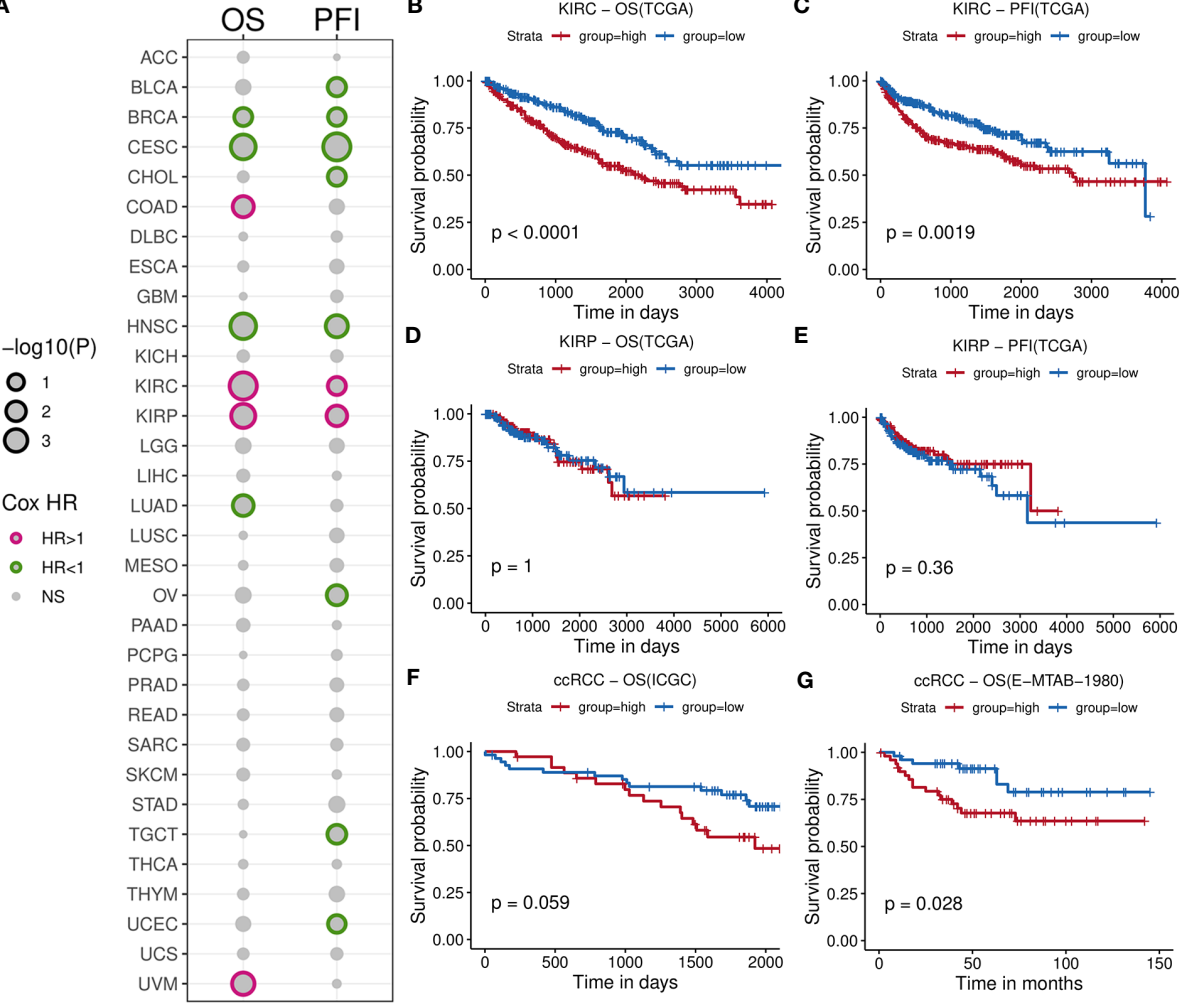
Comprehensive survival analysis for TIL-Bs in pan-cancer and validation of other cohorts for ccRCC. **(A)** Bubble chart illustrating Cox regression analysis of TIL-Bs across 33 TCGA cancers, where rows represent cancer types and columns represent survival events. Point size reflects the significance of the p-value. Points with p-value < 0.1 are outlined in purple (HR > 1) or green (HR < 1). **(B–E)** Kaplan-Meier survival curves showing the relationship between TIL-Bs and survival outcomes in TCGA-KIRC cohort based on OS **(B)** or PFI **(C)** events, and TCGA-KIRP cohort based on OS **(D)** or PFI **(E)** events. **(F, G)** Kaplan-Meier survival curves showing the relationship between TIL-Bs and survival outcomes in the ICGC **(F)** and E-MTAB-1980 **(G)** ccRCC cohorts on OS event.

We initially calculated the HRs of TIL-Bs across 33 types of cancer cells using Cox regression model (**Supplementary Table 1**). As showed in **Figure 2A**, this analysis revealed significant association with negative log10(p-value) less than 1 between TIL-Bs and overall survival (OS) in eight cancers, as well as progression-free survival (PFS) in ten cancers. Notably, two RCC subtypes (KIRC and KIRP) both exhibited the significant HR coefficients above 1 for OS and PFS events. Subsequently, we categorized the two cohort into two groups based on the median infiltration level, respectively. The log-rank test demonstrated higher level of TIL-Bs had indeed lower survival probabilities among KIRC patients (**Figures 2B, C**), while no obvious difference was observed in KIRP cohort (**Figures 2D, E**). Furthermore, two independent ccRCC cohorts also confirmed the noteworthy finding (**Figures 2F, G**). Additionally, from 8 reported markers reflecting B-cell level in tumor environment, some of them, such as PNOC, FCRL2, CD19, were found to have different degrees of impact on poorer prognosis on overall survival of ccRCC cohort (**Supplementary Figure 1A**). The ssGSEA scores of these genes among KIRC patients significantly correlated with B-cell infiltration scores predicted by xCell and display moderate survival difference (**Supplementary Figures 1B, C**). Then, the obvious increasing TIL-Bs was found during the late stage of ccRCC compared to the early stage in both the TCGA and external GSE150404 (**Supplementary Figures 2A, B**) samples. Importantly, two separate survival analysis on early-stage and late-stage patients both showed a certain degree of significance, especially for early-stage patients (**Supplementary Figures 2C, D**).

### 3.2 TIL-Bs related gene modules and ccRCC subcluster

**Figure 3:**
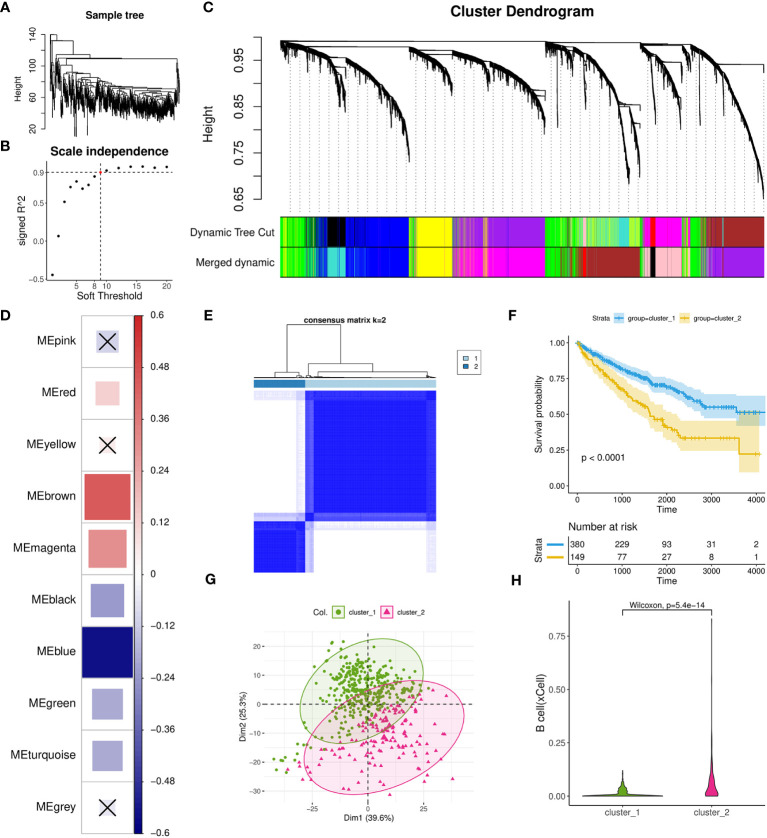
Investigation of TIL-Bs related modules and identification of one ccRCC subtype using WGCNA and Consensus cluster analysis. **(A)** Dendrogram showing the similarity among 529 TCGA-KIRC tumor samples. **(B)** Scatter plot illustrating the relationship between different soft thresholds and scale independence (R2) values. **(C)** Dendrogram of modules generated by WGCNA, representing the hierarchical clustering of genes before and after dynamic merging. **(D)** Heatmap depicting the spearman correlation between ME values and TIL-Bs levels, implemented by the corrplot R package. Cell colors indicate the magnitude of the correlation, where redder cells indicate more positive correlation, and bluer cells indicate more negative correlation. “X” symbols mark cells with p-values > 0.01, which is considered insignificant. Cell size is correlated with absolute correlation coefficient. **(E)** Clustering heatmap generated by ConsensusClusterPlus R package. **(F)** Kaplan-Meier survival curves of two distinct ccRCC subtypes. **(G)** Scatter plot for the result of PCA for the two subtypes. **(H)** Violin plot visualizing the differences in TIL-Bs between the two subtypes.

To investigate the gene networks associated with TIL-Bs in ccRCC, we performed WGCNA to identify co-expression modules using 529 tumor samples from TCGA-KIRC cohort. No considerable outlier sample was detected according to the dendrogram (**Figure 3A**) and the best soft threshold was set to 9 based on relevant topological characteristics (**Figure 3B**) of the expression matrix including 6,000 highly variable genes. Ultimately, ten modules were successfully identified (**Figure 3C**; **Supplementary Table 2**) and their respective hub genes were determined according to module eigengene (ME) values. Then, we calculated the spearman correlation coefficients between TIL-Bs and ME and identified three modules (brown, magenta, red) that exhibited significantly positive correlations (**Figure 3D**). Specifically, brown module exhibited the strongest positive correlation, while blue module exhibited the strongest negative correlation.

Next, we used a total of 431 hub genes of brown and blue modules to execute the resampling consensus algorithm and two distinct ccRCC clusters (cluster_1 and cluster_2) were recognized (**Figure 3E**; **Supplementary Table 3**). PCA based on above genes confirmed clear different expression profile between the two clusters (**Figure 3G**). Through survival analysis, cluster_2 was found to exhibit a significant correlation with worse prognosis outcomes (**Figure 3F**) and also had higher level of TIL-Bs compared to cluster_1 (**Figure 3H**). To gain an understanding of the transcriptomic differences at pathway levels, we firstly applied the DESeq2 tool to identify 1815 DEGs with 1380 upregulated and 435 downregulated (**Supplementary Table 3**), which were used for Reactome pathway enrichment analysis, respectively. The results indicated that the upregulated genes were related to activation of matrix metalloproteinases, GPCR ligand binding and interleukins signaling, while the downregulated gene list had a connection with metabolism of amino acids and derivatives, SLC mediated transmembrane transport and metabolism of vitamins and cofactors (**Supplementary Figures 3A, B**).

### 3.3 Transcriptional characteristics of B cells in ccRCC

**Figure 4:**
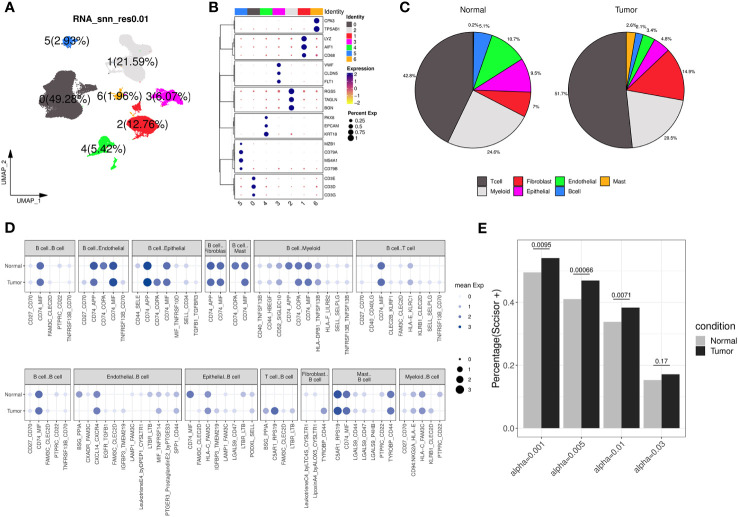
Transcriptomic differences of B cells from a ccRCC scRNA-seq dataset. **(A)** Dimension reduction plot illustrating the distribution of the 7 identified clusters (labelled 0, 1, 2, 3, 4, 5, 6, respectively) under the resolution of 0.01. **(B)** Bubble plot showing the expression of canonical markers across each cluster, where the color and size of the points correspond to the expression levels and percentages, respectively. **(C)** The pie charts displaying the cell type annotation results separately for the normal and tumor groups. **(D)** Bubble plot showing the expression of ligand-receptor pairs in normal and tumor groups, separately. The dot’s size and color indicate the overall mean expression of ligand-receptor partners in corresponding pairs of cell types. If the interaction is not significant according to CellPhoneDB analysis, the expression is considered as 0. Up panel represents B cells as ligands, and down panel represents B cells as receptors. **(E)** Bar chart displaying the percentage of B cells considered as ‘Scissor+’ in the normal and tumor groups under different resolution conditions analyzed by Scissor, with Fisher’s exact test to calculate the distribution difference.

We further examined the transcriptomic changes of B cells in ccRCC tumor. Firstly, a relevant scRNA-seq data (GSE178481) were obtained, of which 10 tumor samples and 9 normal samples were considered. After quality control and batch effect correlation (**Supplementary Figures 4A, B**), we identified 7 clusters at the resolution of 0.01 (**Figure 4A**) and cell type annotation was performed using marker genes (**Figures 4B, C**). In details, cluster 5 exhibited the highest expression of CD79A, CD79B, and MS4A1, indicating its annotation as B cell type. Its proportions in tumor and normal tissue groups were 5.1% and 2.1%, respectively (**Supplementary Table 4**). Utilizing CellPhoneDB analysis, we calculated and observed some altered ligand-receptor interactions involving B cells in the tumor microenvironment (**Figure 4D**). For example, the CD74-COPA pair, from B cells to epithelial cells, was highly expressed in tumor group, while it is not observed in normal group. In addition, we found several activated ligand-receptor interactions, such as C5AR1-RPS19, from T-cell to B-cell of tumor condition. Then, through survival phenotype analysis by Scissor, we found that in tumor condition, there were more B cell subpopulations associated with worse prognosis compared to normal condition, which qualitatively implied TIL-Bs in ccRCC could affect the survival outcomes (**Figure 4E**).

**Figure 5:**
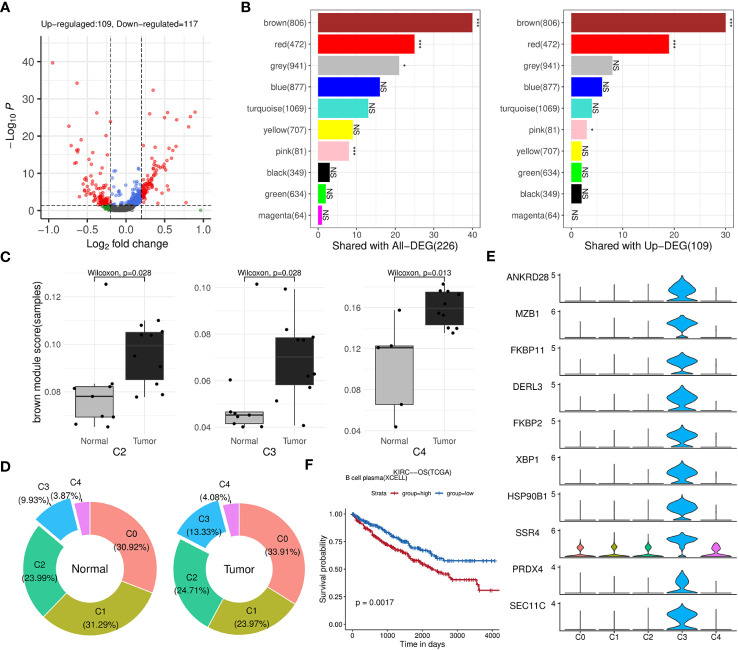
Identification of key modules and related B-cell subpopulations. **(A)** Volcano plot illustrating the differential genes of B cells between the tumor and normal groups. **(B)** Bar charts displaying the number of intersections between each WGCNA module and all differential genes (left panel) or upregulated genes (right panel) in B cells. The significance of the intersection gene proportion was analyzed by Fisher’s exact test. ***p<0.001, *p<0.05, NS p>0.05. **(C)** Box plots illustrating the distribution differences in the brown module scores of the subclusters between the tumor and normal groups at the sequencing sample level. **(D)** Donut charts displaying the composition proportions of the 5 subclusters within B cells in normal (left panel) or tumor (right panel) condition. **(E)** Stacked violin plots showing the expression levels of top 10 differential genes of C5 across five B-cell subclusters. **(F)** Kaplan-Meier survival curves showing the relationship between plasma B-cell and survival outcomes in TCGA-KIRC cohort based on OS event.

Afterwards, we identified 226 genes that were differentially expressed by comparing B cells between the tumor and normal groups (**Figure 5A**; **Supplementary Table 4**). The results of fisher’s exact test showed that brown, red and pink modules was closely related to these DEGs, especially to the upregulated ones (**Figure 5B**). Among the three modules, brown and red modules were also positively correlated with TIL-Bs according to previous analysis, thus considered as core modules related to TIL-Bs in ccRCC. On the other hand, the B cell population was further divided into five subclusters (C0, C1, C2, C3, C4). We assessed the enrichment scores of above two modules among all B cells and the mean scores of each subcluster were compared between tumor and normal condition in the level of sequencing samples. As **Figure 5C** illustrated, there were 3 subclusters displaying significantly higher brown module scores in tumor group. For C3 subcluster particularly, we also found its composition proportion was higher in ccRCC tissues (**Figure 5D**). The subcluster was subsequently annotated as plasma cells due to high expression of markers such as XBP1, CD27 (**Figure 5E**; **Supplementary Table 4**). Log-rank test analysis on TCGA-KIRC cohort implied that the proportion of plasma B cell type was significantly associated with unfavorable overall survival (**Figure 5F**).

### 3.4 Prognostic signature establishment and validation

**Figure 6:**
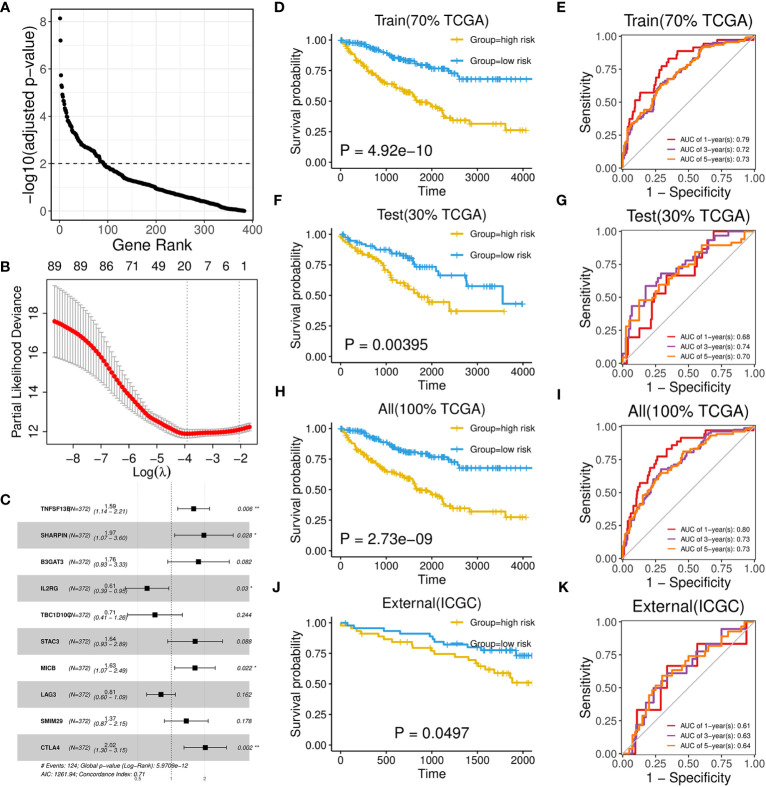
Development and performance of a 10-gene prognostic signature. **(A)** Scatter plot illustrating the ranking of significance in Cox regression analysis for hub genes. **(B)** Scatter plot illustrating the selection of the optimal parameter (lambda) using the ten-time cross-validation method in the Lasso model. **(C)** Forest plot showing the multivariate cox proportional hazards model of the 10-gene signature based on OS event. **(D, F, H, J)** Kaplan-Meier survival curves of two groups divided by median risk score in TCGA train set **(D)**, test set **(F)**, all set **(H)** and ICGC set **(J)** based on OS event. **(E, G, I, K)** Time‐dependent ROC curve analysis of risk score at 1-, 3-, and 5-years OS time in TCGA train set **(E)**, test set **(G)**, all set **(I)** and ICGC set **(K)**.

As brown and red module could represent important roles of B-cell in ccRCC through above analysis, we subsequently aimed to identify potential biomarkers and build a prognostic model from 383 hub genes of the two modules. We randomly divided the TCGA-KIRC dataset into train set and test set in 7:3 ratio (**Table 1**; **Supplementary Table 5**). In the train set, 91 genes with significant HR values of Cox regression analysis (adjusted P value < 0.01) were prioritized (**Figure 6A**) among above hub genes. Using the Lasso model, we further narrowed down the selection to 20 candidate genes with regularization effect (**Figure 6B**). Among them, we found although several genes exhibited moderate predictive performance such as B3GAT3, SHARPIN, the accuracy on external ICGC set were unsatisfactory (**Supplementary Figures 5A–C**). Therefore, through multivariate Cox regression, the combined model with adjusted coefficients of multiple genes from above candidate genes were constructed, and a 10-gene signature (TNFSF13B, SHARPIN, B3GAT3, IL2RG, TBC1D10C, STAC3, MICB, LAG3, SMIM29, CTLA4) was finally proposed (**Figure 6C**). Based risk-score of the signature among the train set, the survival difference between the two groups stratified by the median value cutoff is considerably significant, with the high-score group exhibiting worse prognosis (**Figure 6D**). The AUC values at 1-, 3-, and 5-year overall survival time could reach to 0.79, 0.72, 0.73, respectively (**Figure 6E**). Using the same methods, we assessed the performance of the model in TCGA test set (**Figures 6F, G**) and overall TCGA set (**Figures 6H, I**), which indicated satisfactory efficacy. Importantly, the model also performed well on the ICGC external test dataset, showing its acceptable generalization ability (**Figures 6J, K**).

**Table 1:** The distribution of clinical phenotypes in TCGA-KIRC train and test set.

	Test	Train	Overall
	(N=155)	(N=372)	(N=527)
Gender			
FEMALE	60 (38.7%)	124 (33.3%)	184 (34.9%)
MALE	95 (61.3%)	248 (66.7%)	343 (65.1%)
Age			
Mean (SD)	61.6 (12.7)	60.3 (11.9)	60.7 (12.1)
Median [Min, Max]	63.0 [29.0, 90.0]	60.0 [26.0, 90.0]	61.0 [26.0, 90.0]
OS			
Alive (0)	104 (67.1%)	248 (66.7%)	352 (66.8%)
Dead (1)	51 (32.9%)	124 (33.3%)	175 (33.2%)
Stage			
I	76 (49.0%)	187 (50.3%)	263 (49.9%)
II	17 (11.0%)	39 (10.5%)	56 (10.6%)
III	37 (23.9%)	85 (22.8%)	122 (23.2%)
IV	23 (14.8%)	60 (16.1%)	83 (15.7%)
[Discrepancy]	2 (1.3%)	1 (0.3%)	3 (0.6%)

### 3.5 Aberrant expression of signature biomarkers in ccRCC

**Figure 7:**
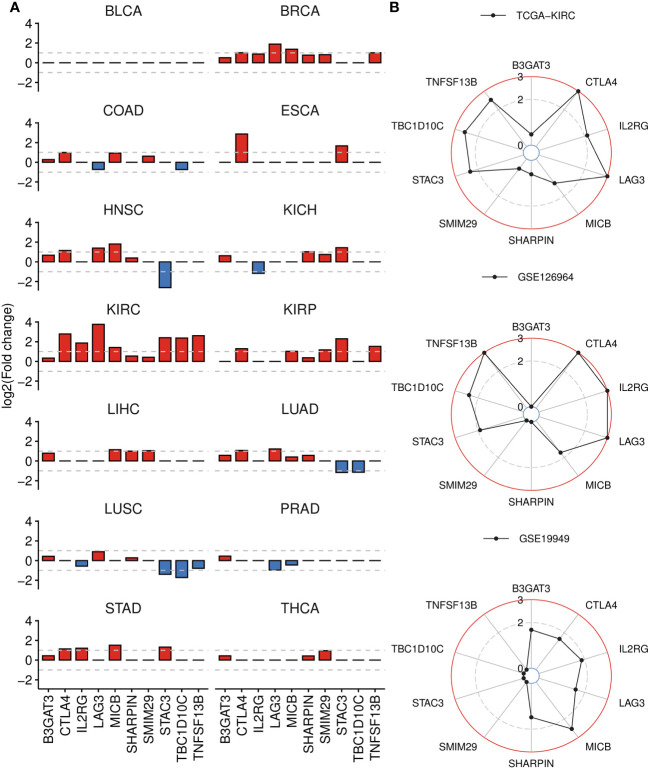
Differential gene analysis of signature genes in TCGA-KIRC and other datasets. **(A)** Bar plots displaying the log2(Fold change) of signature genes between tumor and normal groups across 14 cancer types, derived from GSCA. If the adjusted P-value is greater than 0.01, it is denoted as 0. **(B)** Radar charts displaying the log2(Fold change) of signature genes across three datasets, analyzed by DESeq2 (bulk RNA-seq) or limma (microarray) package. Up panel: TCGA-KIRC dataset, tumor versus normal tissue. Middel panel: GSE126964 dataset, tumor versus normal tissue. Down panel: GSE19949 dataset, metastatic versus primary tissue. If the adjusted P-value is greater than 0.01, it is denoted as 0.

We assessed the differential expression of the 10 signature genes relative to corresponding normal tissues across TCGA cancers and observed significant upregulation of most genes in the KIRC cohort, followed by BRCA, KIRP, HNSC and STAD (**Figure 7A**; **Supplementary Table 6**). Particularly, abnormally higher expression of TBC1D10C was detected exclusively in ccRCC cancer. We further analyzed an Asian cohort (GSE126964), which exhibited similar patterns to those observed in TCGA-KIRC, except for SMIM29, SHARPIN, B3GAT3 (**Figure 7B**). Moreover, six out of ten upregulated genes in ccRCC tumors also displayed elevated expression in metastatic ccRCC samples (GSE19949), such as LAG3 and IL2RG (**Figure 7B**). Interestingly, B3GAT3 and SHARPIN were also found to be highly expressed in this comparison.

**Figure 8:**
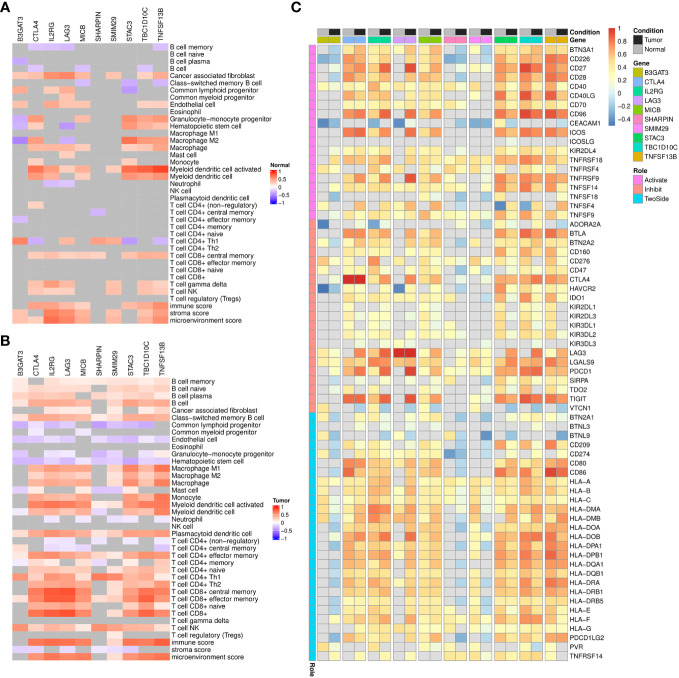
Correlation analysis of signature genes with immune infiltrations, immune checkpoints in TCGA-KIRC dataset. **(A, B)** Heatmap displaying the spearman correlation coefficients between signature genes and immune infiltration scores among TCGA-KIRC normal **(A)** and tumor **(B)** samples, respectively. Grids with p-values above 0.05 in the correlation analysis are colored in gray. **(C)** Heatmap displaying the pearson correlation coefficients between signature genes and immune checkpoints expression among TCGA-KIRC normal or tumor samples. Grids with p-values above 0.05 in the correlation analysis are colored in gray.

Then, through correlation analysis, we revealed that the biomarkers constituting of the prognostic model were unrelated to B-cell infiltration, and a few even show a negative correlation in normal condition (**Figure 8A**). However, their significantly positive correlations were observed with both B-cell and its subtypes in ccRCC tumors (**Figure 8B**). Besides, the correlations with other immune scores were also considerably altered in the tumor environment, such as T cells, Endothelial cells and Monocytes. On the other hand, we also analyzed the relationship with the common ICs (**Figure 8C**). The results indicated seven of them showed obvious association in both conditions and some notable changes were also observed. For example, CTLA4, STAC3, TBC1D10C and TNFSF13B were found to show weaker correlation with KIR (Killer Cell Immunoglobulin-like Receptor) family genes in tumor condition.

### 3.6 Risk-score related clinical phenotypes, SNVs and pathways

**Figure 9:**
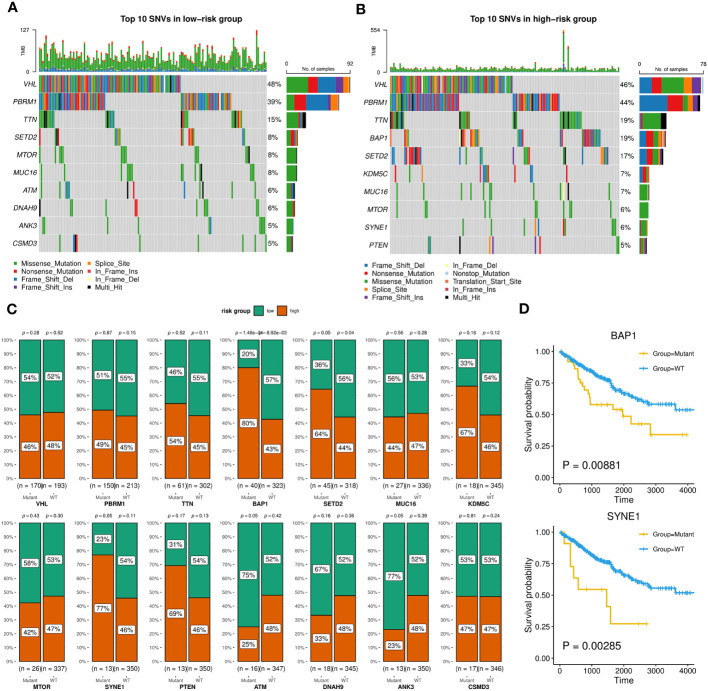
SNVs related to patients in high-risk or low-risk group. **(A, B)** Oncoplots showing the top 10 SNVs of low-risk **(A)** or high-risk **(B)** group, implemented by the maftools R package. **(C)** Bar charts displaying the percentages of high-risk and low-risk patients within the mutant or wild-type groups for each gene, with corresponding p-values of chi-square analysis marked, implemented by the ggstatsplot R package. **(D)** Kaplan-Meier survival curves between wild-type and mutant groups of BAP1 and SYNE1 based on OS event.

Firstly, we evaluated the distribution differences of risk scores across different phenotypes and did not detect significant difference in gender or age grouping (**Supplementary Figure 6**). On the other hand, patients in advanced stage or high grade tended to have higher risk scores (**Supplementary Figure 6**). Then, the KIRC cohort was divided into high-risk and low-risk groups based on the median risk score. Combined with matched SNV data from TCGA, we separately identified top 10 SNVs with the highest mutation frequencies in each group (**Figures 9A, B**), which existed some distinctions, such as BAP1, ATM, SYNE1. We furtherly employed Chi-squared test to compare the number of patients in high-risk and low-risk within the wild group or mutant group of above genes. As **Figure 9C** showed, the mutations of BAP1, SED2, SYNE1 were found to be more closed to high-risk patients. In particular, two of them (BAP1, SYNE1) were also related to worse survival (**Figure 9D**).

**Figure 10:**
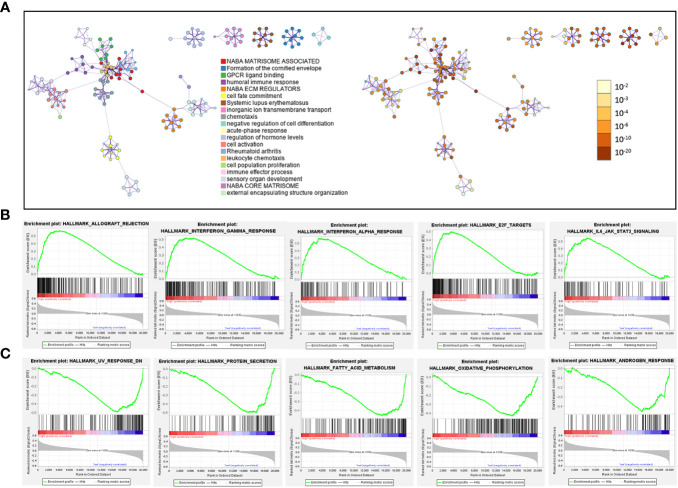
Pathways related to difference between patients in high-risk and low-risk groups. **(A)** Network diagram, where each term is depicted as a circle and its size reflecting the number of associated input genes. Node color denotes cluster identity (left panel) or p-value (right panel), while edges connect terms with similarity scores > 0.3 (edge thickness indicates similarity) **(B, C)** GSEA plot showing the top 5 pathways, with most positive **(B)** or negative **(C)** enrichment scores, in the high-risk group compared to the low-risk group.

Next, we identified 1258 significant DGEs between the two risk groups, which were used to annotate the twenty most associated functions by the Metascape platform (**Figure 10A**; **Supplementary Table 7**). The enrichment results mainly involved extracellular matrix and matrisome, GPCR (G protein-coupled receptor) ligand binding, humoral immune response, cell population proliferation, cell fate commitment. Through GSEA analysis based on hallmark pathways from MSigDB database (**Supplementary Table 7**), we found 12 upregulated terms including IFN-alpha response, IFN-gamma response, IL-6/JAK/STAT3 signaling (**Figure 10B**) and 14 downregulated terms including protein secretion, fatty acid metabolism, androgen response (**Figure 10C**).

### 3.7 General nomogram model based on risk score, stage and age of patients

**Figure 11:**
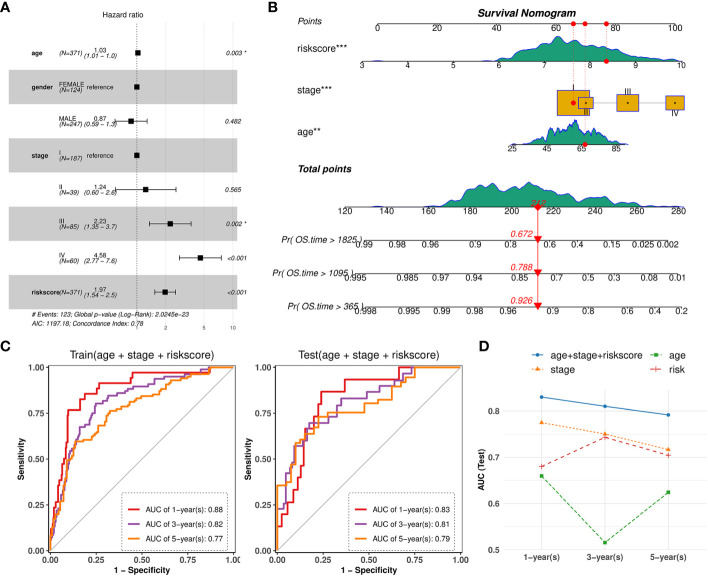
Nomogram combined risk-score and other clinical phenotypes. **(A)** Forest plot showing the multivariate Cox regression model of the risk score and other clinical phenotypes. **(B)** Nomogram for predicting survival probability by integrating risk score, tumor stage, patient age. Each factor is assigned a score contributing to the total point assessment. **(C)** Time‐dependent ROC curve analysis of the nomogram at 1-, 3-, and 5-years OS time in TCGA-KIRC train set and test set. **(D)** Line charts depicting the AUC values at 1-, 3-, and 5-years OS time for the nomogram and three individual factors.

Based on the validated prognostic value of the 10-gene signature, we further confirmed its independent contribution to survival prediction while adjusting for other potential covariates such as age, sex, and stage. The results of multivariable Cox regression analysis demonstrated that the risk score, patient age and AJCC stage, with hazard ratios above 1, had significant effects on survival outcomes (**Figure 11A**). The overall concordance index (C-index) reached 0.78, indicating good predictive performance in the train set. Consequently, an intuitive nomogram was established by summing up the scores for above three factors (**Figure 11B**). Then, AUC values at 1-year, 3-years, 5-years of time points based on the nomogram were evaluated on TCGA-KIRC train (0.88, 0.82, 0.77) and test set (0.83, 0.81, 0.79), which both showed significant efficacy (**Figure 11C**). Importantly, the predictive performance of the combined model was found to be superior to that of simple model based on any individual factor (**Figure 11D**).

### 3.8 Potential therapeutic drugs against high-risk patients

Finally, we intended to discover potential therapeutic drugs for ccRCC patients with higher risk scores based on the TIL-Bs related signature. Combining with the predicted IC50 data of 274 drugs across TCGA-KIRC patients, we calculated their pearson correlation coefficients with risk scores. Then, 38 compounds that display strong negative correlation (R < -0.3) were screened. For the top 10 of them (**Table 2**), we found high-risk group patients are more sensitive with significantly low drug IC50 compared that of low-risk group, such as AZD6738, AICA Ribonucleotide, Bosutinib (**Supplementary Figure 7**).

**Table 2:** The top 10 drugs most negatively correlated with the risk-score.

Rank	Name	PubChem CID	Molecular Formula	R (pearson)	P.value	Target	Target.pathway
1	AZD6738	54761306	C20H24N6O2S	-0.578664723	1.60E-48	ATR	Genome integrity
2	AICA Ribonucleotide	65110	C9H15N4O8P	-0.513031165	8.74E-37	AMPK agonist	Metabolism
3	Bosutinib	5328940	C26H29Cl2N5O3	-0.468464746	3.70E-30	SRC, ABL, TEC	Other, kinases
4	MK-8776	16224745	C15H18BrN7	-0.425603419	1.21E-24	CHEK1, CHEK2, CDK2	Cell cycle
5	Tubastatin A	49850262	C20H21N3O2	-0.425040135	1.41E-24	HDAC1, HDAC6, HDAC8	Chromatin histone acetylation
6	Ulixertinib	11719003	C21H22Cl2N4O2	-0.422751508	2.65E-24	ERK1, ERK2	ERK MAPK signaling
7	GSK343	71268957	C31H39N7O2	-0.420283431	5.18E-24	EZH2	Chromatin histone methylation
8	BIX02189	135659062	C27H28N4O2	-0.419452228	6.49E-24	MEK5, ERK5	ERK MAPK signaling
9	Cyclopamine	442972	C27H41NO2	-0.405539225	2.55E-22	SMO	Other
10	AZD7762	11152667	C17H19FN4O2S	-0.389473139	1.43E-20	CHEK1, CHEK2	Cell cycle

## 4 Discussion

The TME in solid tumors is primarily composed of various immune cells, including T cells, B cells, macrophages, dendritic cells, natural killer cells, and neutrophils. While B cells are recognized as significant contributors to humoral immunity and have been extensively studied in the context of autoimmune diseases, their precise roles within cancer remain incompletely understood, partly due to their relatively lower abundance in the TME (35, 36). Furthermore, a few studies have investigated the potential anti-tumor response of TIL-Bs considering their potential ability to secrete immunoglobulins or support T cell responses in the fight against cancer (37). However, it has been reported that TIL-Bs in ccRCC could have a different impact on patient survival, and previous studies have not fully explored their multifaceted features. Therefore, it is worthwhile to conduct a systematic investigation into the prognostic significance and molecular characteristics of TIL-Bs on ccRCC tumors and the underlying mechanisms, which can provide further insights into the complex tumor microenvironment in ccRCC and facilitate the development of novel treatment strategies.

First of all, B-cell infiltration levels of TCGA-KIRC cohort predicted by xCell algorithm were confirmed to be associated with worse survival outcomes in OS and PFI events of patients among 33 TCGA cancer types. The finding was further validated by another two ccRCC cohorts and several TIL-Bs related markers. Next, we identified 10 co-expression modules in ccRCC tumor tissues using the WGCNA method, and calculated their correlation with TIL-Bs to uncover potential gene regulatory networks. Among three positively related modules (brown, magenta, red) and four negatively modules (blue, black, green, turquoise), the brown and blue modules showed remarkable correlation, respectively. Based on hub genes of the two modules, we performed consensus cluster analysis, revealing a distinct KIRC subtype characterized by high B-cell infiltration level and poor prognosis. Furthermore, the upregulated and downregulated pathways of this subtype were annotated by corresponding DEGs. According to the results, we inferred an underlying extracellular matrix (ECM) remodeling process in cluster_2 subtype, which could influence the recruitment and activity of immune cells (38). Besides, both interleukins signaling and NF-κB pathway can also participate in B-cell regulation and function (39–41).

On the other hand, we sought to explore the underlying relationship of above modules to the transcriptomic changes of B cells in tumor environment. Based on a public ccRCC scRNA-seq dataset, we annotated a cluster of over 3,000 cells with higher expression of B-cell markers such as CD79A, CD79, and MS4A1, and furtherly identified five subpopulations. Firstly, cell communication and Scissor phenotype analysis revealed that B cells in tumor group indeed played a different role in the interaction with other cell types and would be unfavorable for patient survival, which both suggested that the unignorably altered expression of TIL-Bs were existed. Consequently, we found three modules (brown, red, pink) that were considerably close to the DEGs of TIL-Bs. Noteworthily, brown and red modules showed significantly positive correlation with B-cell infiltration in previous analysis. Therefore, the brown module with 806 genes and red module with 472 genes could be the two core networks which helped us to understand the characteristic of TIL-Bs and explain their impact on survival prognosis of ccRCC patients.

Additionally, we also investigated the B-cell subclusters through module scoring analysis and 3 of them showed increasing expression score for above brown module in tumor samples. For C3 subcluster in tumor group particularly, we found it was amplified compared to that in normal group. Then, we designated this subcluster as plasma B cells due to its specific expression of CD27, IGJ, XBP1, etc. and survival analysis confirmed this subtype was also related to worse survival in KIRC cohort. Previous research have found TIL-Bs exhibited general phenotypes including naïve, activated and memory B cells, germinal center B cells, and plasma cells (42). Among these phenotypes, plasma cells, which are responsible for antibody production, represent one of the final stages of B-cell development. In a study on non-small cell lung cancer (NSCLC), plasma B cells were found to play contradictory roles at different stages of NSCLC (43).

Based on the two core modules through above analysis, our consequent objective was to narrow down the potential biomarkers associated with TIL-Bs in ccRCC and build a relevant prognostic model. Initially, we divided the TCGA-KIRC cohort into train set and test set, with the ICGC cohort serving as an external test set. Through univariate Cox regression analysis and Lasso model analysis on the training set, we screened 20 candidate molecules from 383 hub genes of the brown and red module. Ultimately, a 10-gene signature was proposed and the risk score calculated based on the model showed good predictive performance on train set and test set from TCGA samples, with higher values indicating worse survival outcomes. Specially, we also found its generalization ability on independent ICGC set were satisfactory and better than that of most single candidate gene.

Through pan-cancer analysis, the 10 markers composed of the signature were found to show varying degrees of upregulation in ccRCC. Among them, some genes have been confirmed to be associated with the development and proliferation of B cells. For example, TNFSF13B (TNF Superfamily Member 13b) is responsible for encoding a cytokine, named BAFF (B cell-activating factor), which belongs to the tumor necrosis factor (TNF) family (44). It has been known as important ligand for 3 receptors (BCMA, TACI, BAFF-R) expressed by B cells and consequently participate in B-cell differentiation, proliferation, survival, and functional responses (45, 46). One clinical research recently reported that BAFF significantly associated with worse OS in patients with metastatic ccRCC (47). Regarding to SHARPIN (SHANK Associated RH Domain Interactor), we found that although its expression alteration was not remarkable in primary tumor tissues, it was significantly upregulated in metastatic ccRCC. Its encoding protein was one of essential subunits to form the linear ubiquitin chain assembly complex (LUBAC) (48, 49). Previous researches found there is a diminished activation of the IKK complex and NF-κB in B cells in mice with SHARPIN deficiency (49) and LUBAC could involve in B-cell activation by regulating CD40 signaling (50). Another report indicated knockdown of SHARPIN could inhibit ccRCC tumor growth in xenograft models (51). TBC1D10C (TBC1 Domain Family Member 10C), also named Carabin, is an inhibitor of both the Ras signaling pathway and calcineurin (52). We found it showed exclusively upregulation in ccRCC in our pan-cancer analysis. Interestingly, the gene exhibited a significant HR value above 1. However, in the combined signature model, its adjusted HR was below 1, meaning an oppositely protective role. Recent research found it indeed could be a key suppressor of B-cell receptor signaling and proliferation via Ras/extracellular signal-regulated kinase pathway in B-cell lymphoma (53).

Afterwards, we calculated the correlation of the signature genes with common immune infiltration scores and immune checkpoint genes, and compared the differences between normal and tumor tissues. As expected, we observed the significant positive correlation with B-cell infiltration in tumor tissue. Moreover, CD8+ T cells related scores also showed notably increasing correlation with most of the 10 genes, which played important role in tumor immunotherapy (54). In addition, 2 (CTLA4 and LAG3) of the 10 genes belong to classical immune checkpoints, which could contribute to tumor cells in evading immune surveillance (55). Consequently, we also explored the links to common ICs using the same way. According the result, more than half of the signature genes showed significantly positive correlations on both conditions. For the remain three genes had little relationship in normal group and slightly negative correlation in tumor group. Above findings revealed that the signature was not only related to TIL-Bs but also had underlying links with CD8+ T cells mediated immune response to ccRCC.

Next, the overall characteristics of the ILB-related signature were also examined. Firstly, we observed that patients with higher risk scores were often found at advanced stages, which aligned with the primary survival analysis. Furthermore, we identified several SNVs which were related to patients in high-risk group. Two (BAP1 and SYNE1) of them showed worse survival compared to their wild type. In particular, of 40 patients with BAP1 mutation, 80% were in high-risk group, while previous study found that it was recurrent in both earlier and advanced ccRCC (56). Another research on melanoma illustrated the absence of BAP1 correlates with an increase in the activity of the NF-κB pathway (57). Through pathway analysis based on the transcriptomic difference between high-risk and low-risk patients, we revealed potential related processes such as extracellular matrix which was similar with the characteristics of above TCGG-KIRC subtype identified by TIL-Bs related modules. Besides, we also observed the enrichment for cell population proliferation and B-cell mediated humoral immune response. Furthermore, the upregulation of inflammation-related pathways was detected, such as IL6/JAK/STAT3 signaling, interferons-alpha (IFN-α), and interferon-γ (IFN-γ) response. Additionally, we found a negative enrichment of androgen receptor (AR) response, which has been shown in previous research to suppress the development and activation of B cells (58).

As preliminary survival analysis has indicated the relationship of TIL-Bs with tumor stage, therefore, we aimed to build a comprehensive model by merging the gene signature and common clinical phenotypes. Ultimately, we established a nomogram based on three independent factors (risk score, tumor stage and patient age) and its AUC performance on test set generally increased to 0.8. On the other hand, we also screened some potential drugs that displayed more sensitive IC50 values on high-risk group. Among the top 10 drugs, AICA Ribonucleotide (AICAR) was known as an activator of AMPK, which has been considered as a possible target for RCC (59, 60). Recent two studies also found AMPK could also could inhibit NF-κB activation (61, 62). In addition, according to a preclinical study, the combination of AICAR and rapamycin has been reported to slow down the progression of kidney tumors (63).

In conclusion, our study uncovered the prognostic and transcriptomic characteristics of TIL-Bs in ccRCC, revealing their association with poor survival, which distinguishes them from most other cancers. Through gene co-expression network analysis, we identified several modules associated with TIL-Bs and consequently detected one unique cluster of ccRCC patients and one abnormal TIL-Bs subcluster based on several key ones. By considering two core modules, we ultimately constructed a prognostic signature consisting of 10 potential biomarkers. We thoroughly explored and discussed the key features of both individual signature genes and the signature as a whole at multiple levels. Furthermore, we investigated the potential clinical applications of the signature, including nomogram survival prediction and drug discovery, which yielded promising results. It is also important to acknowledge the limitations of our research. Further in-depth biological interpretation of our findings is warranted, and wet laboratory experiments are essential for future validation. Clinical assessments of the proposed signature are needed to evaluate its potential in guiding treatment decisions and patient management. Nonetheless, our study offers valuable insights into the roles of TIL-Bs in ccRCC.

## Data availability statement

The original contributions presented in the study are included in the article/**Supplementary Material**, further inquiries can be directed to the corresponding author.

## Author contributions

SL and YY conceived and designed the study. XC and CL collected the data. SL and XC analyzed and interpreted the data. YY, CL, SL drafted the manuscript, and all authors critically revised it for intellectual content. SL, YY, LS, PS participated in manuscript revision process. All authors have read and approved the final version of the manuscript.
