# Supplementary material for: Unraveling the prognostic significance and molecular characteristics of tumor-infiltrating B lymphocytes in clear cell renal cell carcinoma through a comprehensive bioinformatics analysis
Source: Front Immunol. 2023 Oct 16;14:1238312. doi: 10.3389/fimmu.2023.1238312 (PMC10613680; doi:10.3389/fimmu.2023.1238312)
Supplement: Supplementary file 1 [file DataSheet_1.docx]

Supplementary Material

Unraveling the Molecular Characteristics and Prognostic Significance of Tumor-Infiltrating B Lymphocytes in Clear Cell Renal Cell Carcinoma through a Comprehensive Bioinformatics Analysis

**Youwei Yue^1^, Xinyi Cai^2^, Changhao Lu^3^, Leonardo Antonio Sechi^3^, Paolo Solla^4^, Shensuo Li^5^**

^1^Department of Urology, Longgang District Central Hospital of Shenzhen, Shenzhen, China

^2^Department of Pathology, Provincial Key Laboratory of Infectious Diseases and Molecular Immunopathology, Shantou University Medical College, Shantou, China

^3^Department of Biomedical Sciences, University of Sassari, Sassari, Italy

^4^Department of Medical, Surgical and Experimental Sciences, University of Sassari, Sassari, Italy

^5^Shanghai Frontiers Science Center for Chinese Medicine Chemical Biology, Institute of Interdisciplinary Integrative Medicine Research, Shanghai University of Traditional Chinese Medicine, Shanghai, China;

*** Correspondence:**Shensuo Li
[lishensuo@163.com](mailto:lishensuo@163.com)

# Supplementary Figures and Tables

## Supplementary Figures


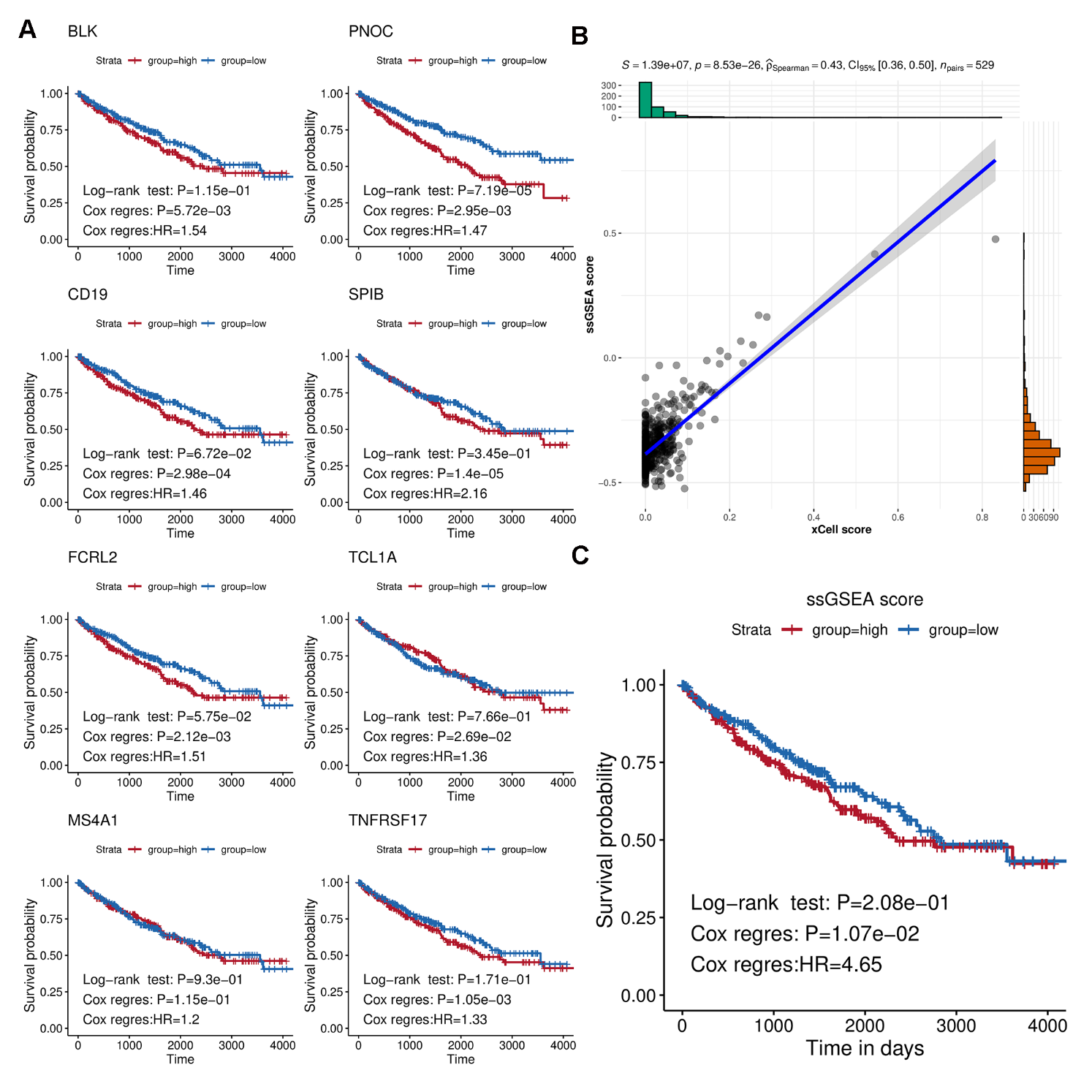


**Supplementary Figure 1** | Survival analysis for TIL-Bs related markers and ssGSEA score in TCGA-KIRC cohort. **(A)** Kaplan-Meier survival curves showing the relationship between eight TIL-Bs related markers and survival outcomes in TCGA-KIRC cohort based on OS event. **(B)** Scatter plot depicting the correlation between ssGSEA expression scores of eight markers and xCell infiltration score of TIL-Bs, implemented by the ggstatsplot R package. The spearman correlation coefficient and its p-value were marked. **(C)** Kaplan-Meier survival curves showing the relationship between ssGSEA scores and survival outcomes in TCGA-KIRC cohort based on OS event.


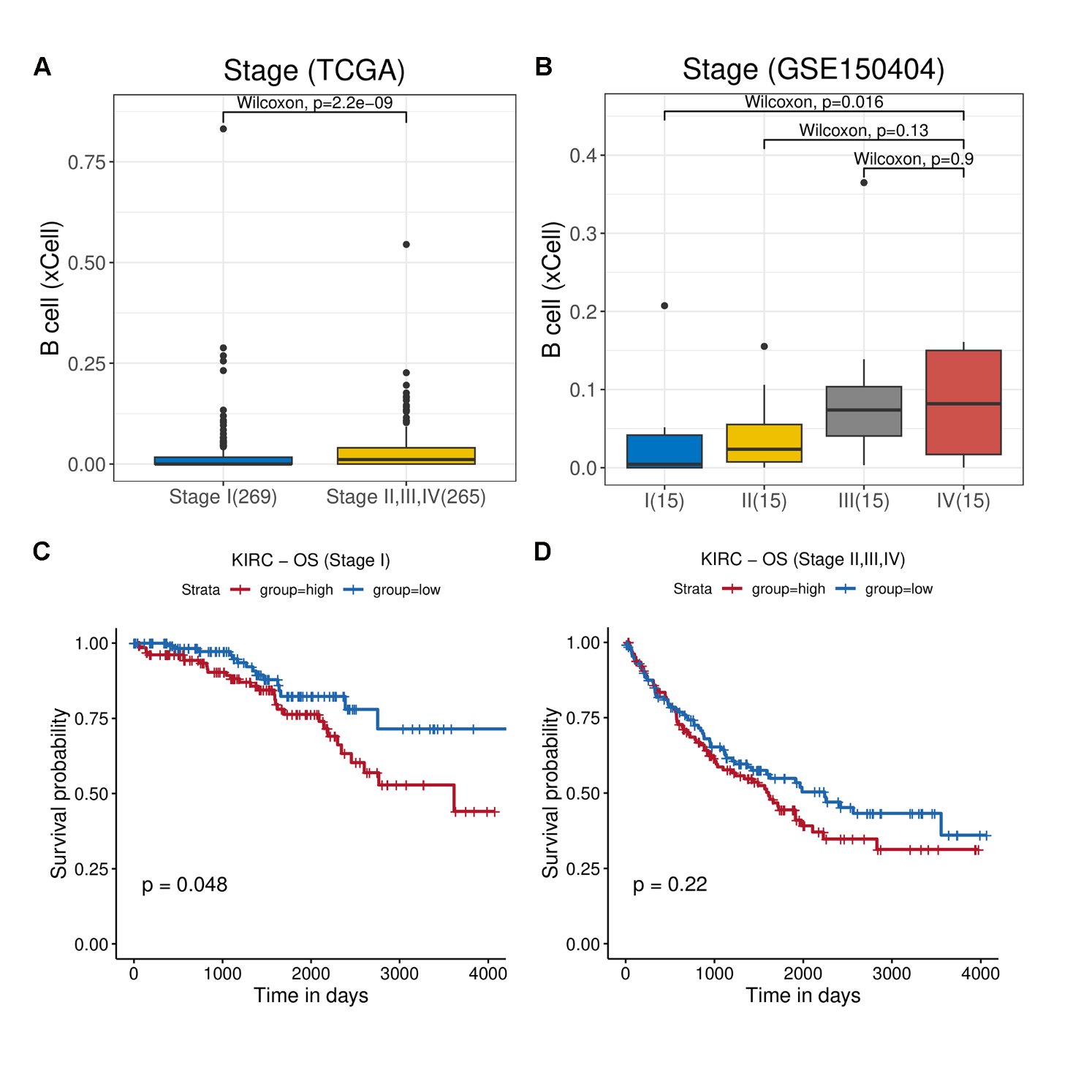


**Supplementary Figure 2** | TIL-Bs differences and survival analysis in among different stages of ccRCC patients. **(A-B)** Box plot visualizing the differences in TIL-Bs levels among different tumor stages of TCGA-KIRC **(A)** and GSE150404 **(B)** cohorts. **(C-D)** Kaplan-Meier survival curves showing the relationship between different tumor stages and survival outcomes in TCGA-KIRC cohort based on OS event. **(C)** for early stage; **(D)** for late stage. In details, the median value was used for dividing into high- and low-level TIL-Bs.


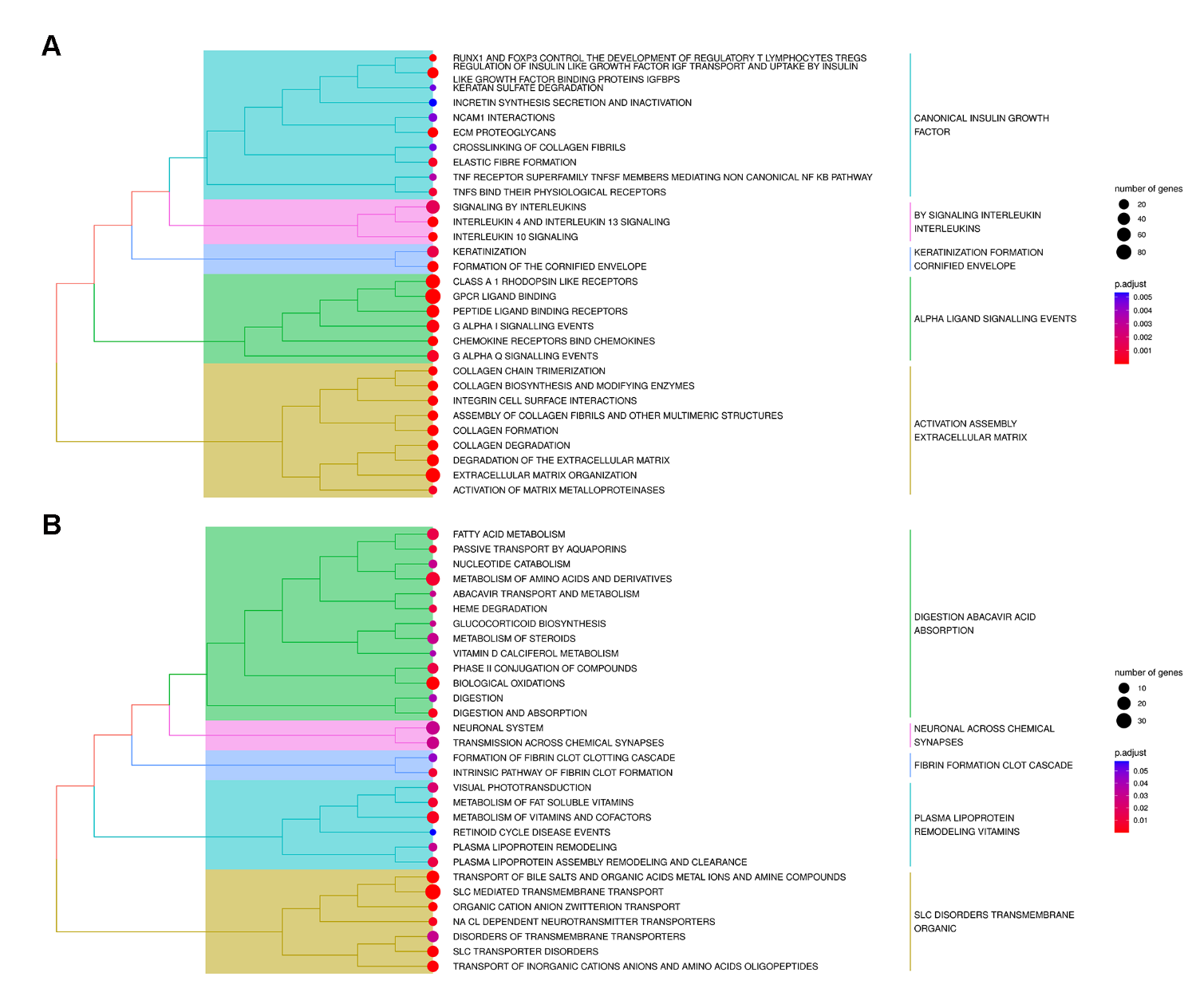


**Supplementary Figure 3** | Reactome pathway enrichment for DEGs between TCGA-KIRC subtypes. **(A)** for upregulated DEGs; **(B)** for downregulated DEGs. The tree diagrams were visualized by enrichplot R package, where 5 term clusters were divided and 4 key words were extracted for each cluster.


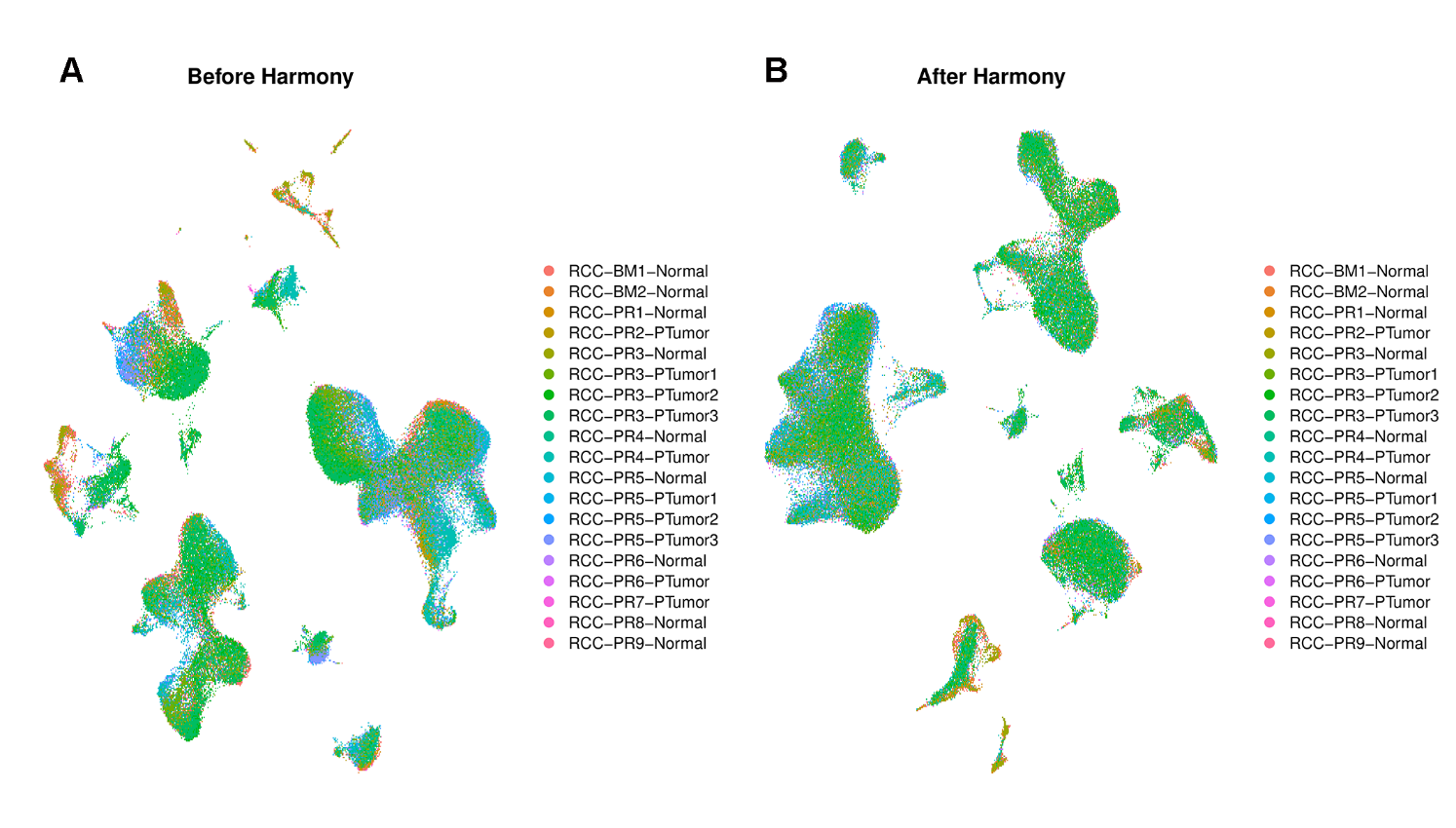


**Supplementary Figure 4** | UMAP dimension reduction plot displaying the distribution of cells from different sample sources before **(A)** and after **(B)** harmony batch correction.


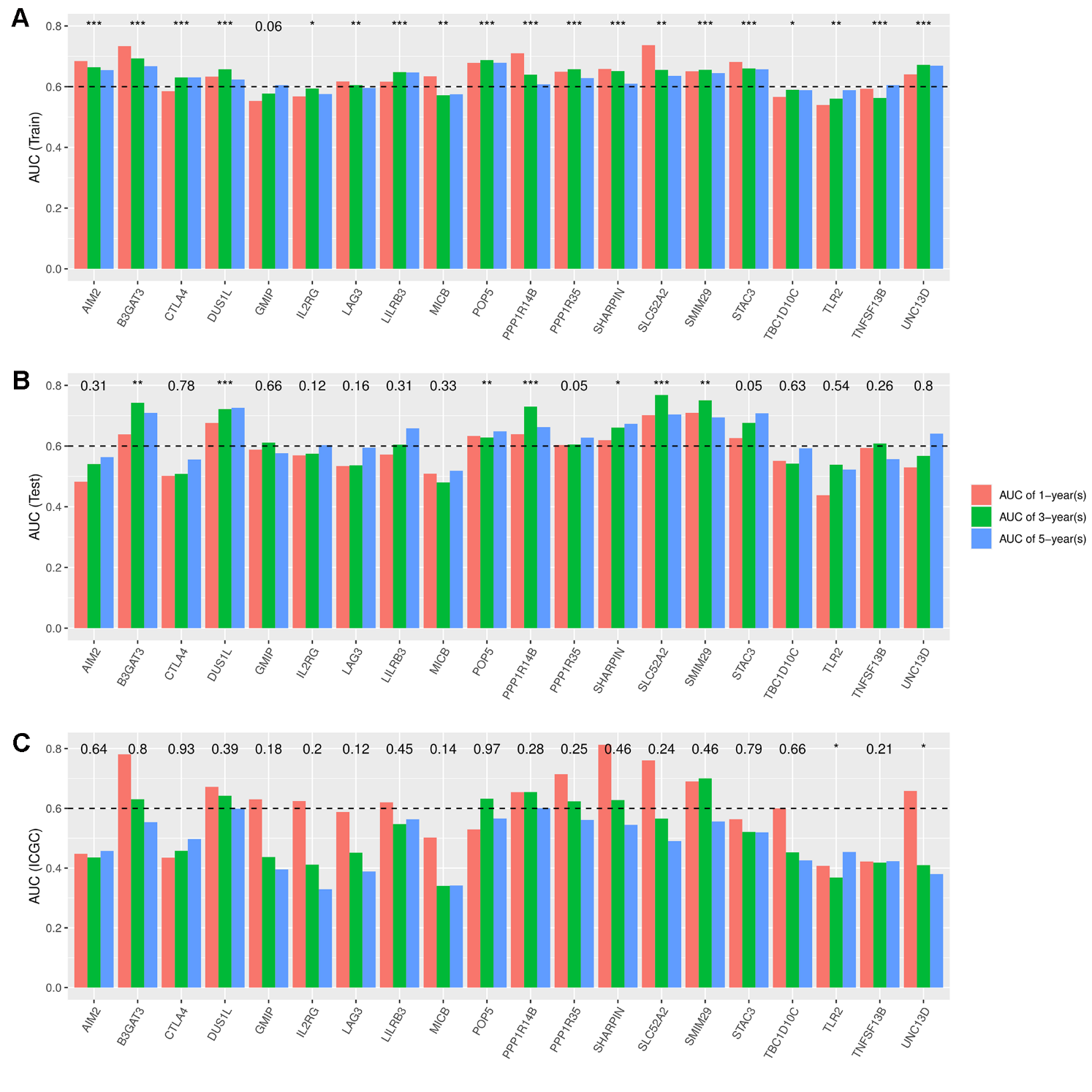


**Supplementary Figure 5** | Bar plots showing AUC values at 1-, 3-, and 5-year OS time of 20 candidate genes in TCGA train set **(A)**, test set **(B)** and ICGC set **(C)**. The significance of log-rank test based the grouping of median expression level was labeled. * P<0.05, ** P<0.01, *** P<0.001.


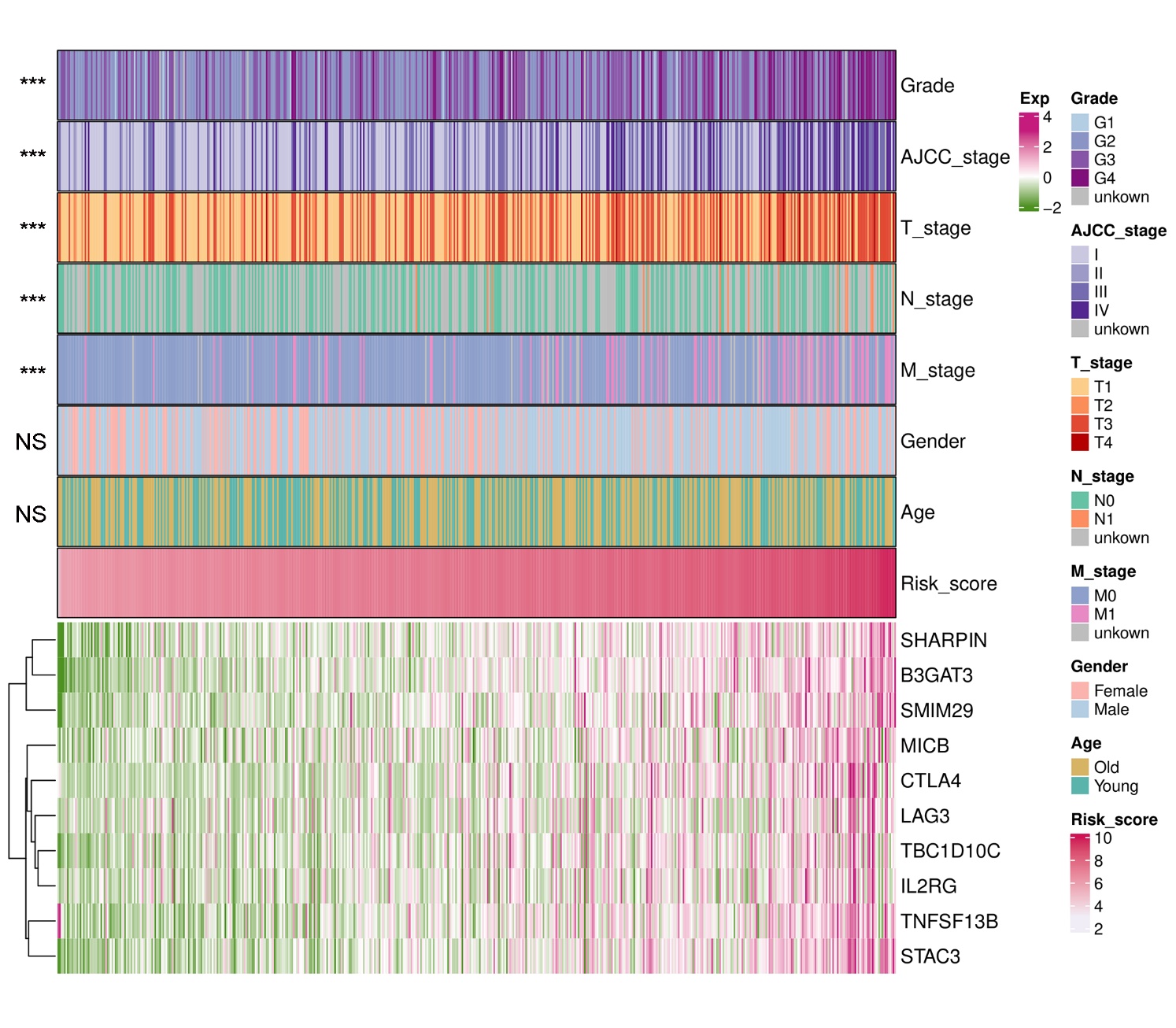


**Supplementary Figure 6** | Heatmap displaying the expression of signature genes in the KIRC cohort, with samples sorted from left to right in ascending risk scores. The top annotation corresponds to five clinical phenotypes for the samples, and an analysis of variance (ANOVA) was performed to assess the differences in risk scores among these groups. NS P>0.05, *** P<0.001. For age, the samples were divided into two groups (old and young) based on a threshold of 60.


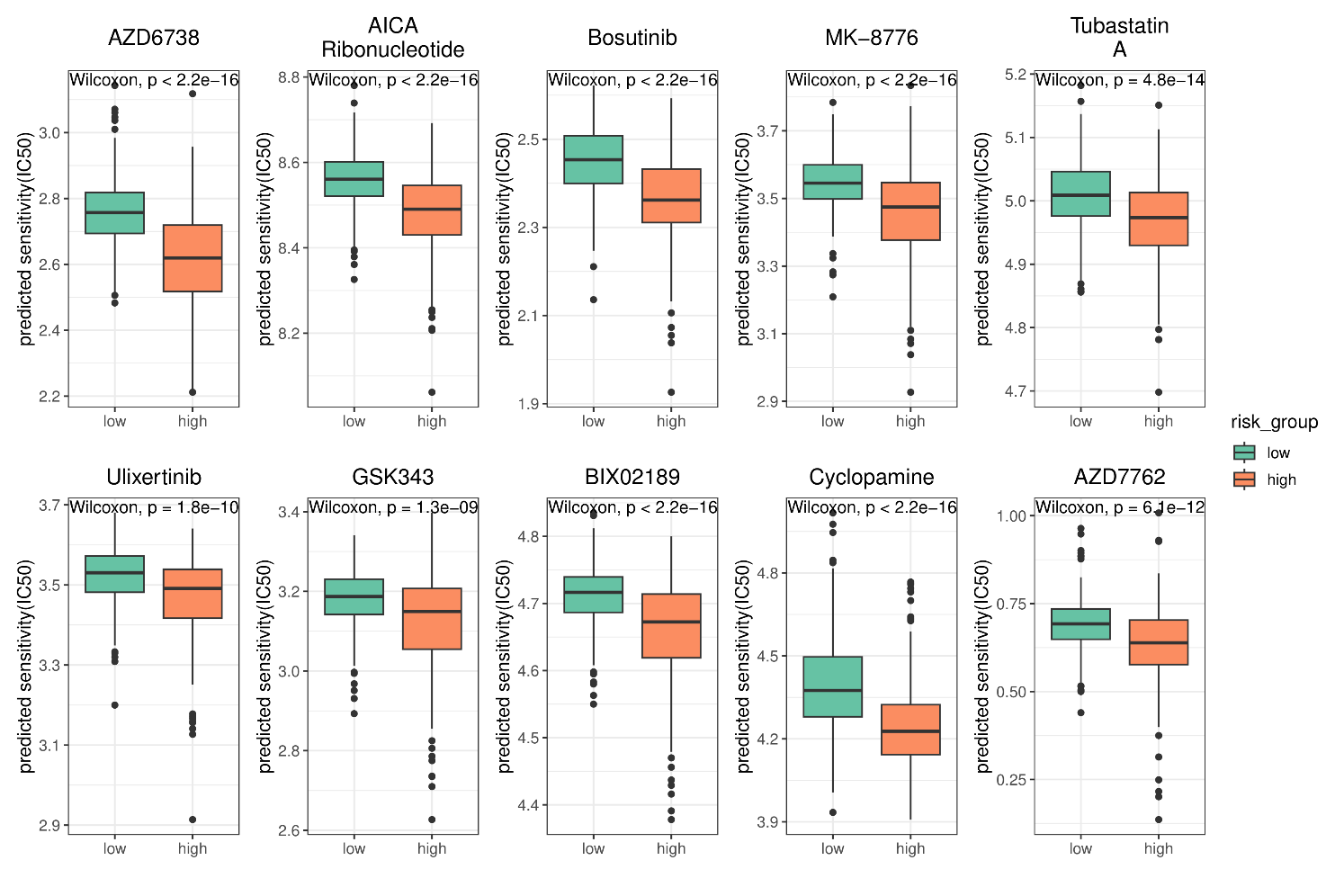


**Supplementary Figure 7** | Box plots displaying the differences in sensitivity distributions of 10 drugs between high-risk and low-risk groups.

## Supplementary Tables

**Supplementary Table 1**: TIL-Bs scoring and related survival data, clinical stage for 33 TCGA tumor samples.

**Supplementary Table 2**: The 10 co-expression modules identified by WGCNA.

**Supplementary Table 3**: The two subgroups identified by Consensus cluster analysis and differential genes between them.

**Supplementary Table 4**: The analyzed information of B-cell from a ccRCC scRNA-seq dataset, including cell annotation, Scissor results, CellPhoneDB results and two types of differential genes.

**Supplementary Table 5**: Train and test set from TCGA-KIRC tumor samples, together with their risk score, expression of signature genes.

**Supplementary Table 6**: Differential expression of signature genes between tumor group and normal group at different conditions.

**Supplementary Table 7**: Pathway enrichment between two groups in low-risk and high-risk by Metascape and GSEA tools.
